# Sexual conflict over the duration of copulation in *Drosophila montana*: why is longer better?

**DOI:** 10.1186/1471-2148-9-132

**Published:** 2009-06-12

**Authors:** Dominique Mazzi, Jenni Kesäniemi, Anneli Hoikkala, Kirsten Klappert

**Affiliations:** 1 Department of Biological and Environmental Science, University of Jyväskylä, PO Box 35, FIN-40014, Finland; 2 ETH Zurich, Institute of Plant Sciences, Applied Entomology, Schmelzbergstrasse 9, CH-8092 Zurich, Switzerland; 3 Evolutionary Biology, Dyer's Brae House, University of St Andrews, St Andrews, Fife, KY16 9TH, UK; 4 Aquatic Ecology, EAWAG/ETH, Ueberlandstrasse 133, CH-8600 Duebendorf, Switzerland

## Abstract

**Background:**

Conflicts of interest between the sexes are increasingly recognized as an engine driving the (co-)evolution of reproductive traits. The reproductive behaviour of *Drosophila montana *suggests the occurrence of sexual conflict over the duration of copulation. During the last stages of copulation, females vigorously attempt to dislodge the mounting male, while males struggle to maintain genital contact and often successfully extend copulations far beyond the females' preferred duration.

**Results:**

By preventing female resistance, we show that females make a substantial contribution towards shortening copulations. We staged matings under different sex ratio conditions, and provide evidence that copulation duration is a form of male reproductive investment that responds to the perceived intensity of sperm competition as predicted by game theoretical models. Further, we investigated potential benefits to persistent males, and costs to females coerced into longer matings. While males did not benefit in terms of increased progeny production by protracting copulation, female remating was delayed after long first copulations.

**Conclusion:**

Copulation time is a trait subject to sexual conflict. Mating durations exceeding female optima serve males as a form of 'extended mate guarding': by inducing mating refractoriness in the female, a male extends the time over which its sperm is exclusively used to sire progeny and reduces the likelihood of the female being reinseminated by a competitor.

## Background

While sexual selection has long been regarded as the engine responsible for the co-evolution of male traits and female preferences [1], antagonistic co-evolution between the sexes has only recently gained growing attention (reviewed in [2]). Evolutionary conflicts of interest between the sexes are common, and increasingly recognized as a powerful force driving the evolution of traits involved in reproduction [3-6]. Sexual conflict is expected to fuel the evolution of adaptations that bias the outcome of reproduction towards one sex, even at the expense of the fitness interests of the other. In response, the latter sex should evolve countermeasures that effectively minimize the costs of sexual interactions. Heightened resistance in turn promotes further persistence, leading to an escalating co-evolutionary arms race similar to the Red Queen dynamics in parasite-host relationships [7]. The intraspecific conflict between males and females arises for control over aspects of reproduction such as mating rate, female propensity to remate, female proximate fecundity, or relative parental effort (reviewed in [4]).

The optimal outcome of reproductive interactions for males and females rarely, if ever, coincides [8]. In promiscuous species where sperm competition occurs, males can enhance their reproductive success by either increasing the number of inseminated partners or by preventing their partners from remating. The two strategies are to some extent mutually exclusive, since the time and energy budgeted on a given mating will be traded against the acquisition of further matings [9]. Hence, whenever males incur nontrivial mating costs, they should prudently partition reproductive effort over a series of matings to maximize lifetime reproductive success (reviewed in [10]). The operational sex ratio (OSR, the ratio of males to females ready to mate, [11]) is a reliable predictor of the probability that a male will have to outcompete sperm of rival males to fertilize a female's eggs. While reproductive investment is predicted to rise linearly with the degree of sperm competition across species and indeed does so in several groups (e.g. [12-16]), a different pattern is predicted within species. Within species, a male's expenditure should not only depend on the risk of sperm competition (i.e. competitors present or not) but also on the intensity of competition (i.e. the number of competitors). Parker et al's [17] series of evolutionarily stable strategy models predicts that males should invest little when there are no competitors and most in the presence of one competitor. Somewhat counter intuitively, as the number of competitors exceeds two the models predict a corresponding decrease in expenditure because the marginal gains from any additional unit of investment decrease. Empirical tests have provided some, albeit not ubiquitous, support for a curvilinear relationship between competition intensity and male investment ([18,19] but see e.g. [20]). When facing competition in fertilising eggs, males of some species have been found to prolong copulation duration, e.g. [21].

Long-lasting copulations are widespread in insect species, despite associated potential costs in terms of time, energy, increased predation vulnerability and disease transmission [15,22]. Two main hypotheses, 'the ejaculate transfer hypothesis' and 'the extended mate guarding hypothesis', have been put forward to explain the advantage of protracted mating associations. The 'ejaculate transfer hypothesis' presupposes a more or less linear relationship between copulation duration and sperm transfer and storage, and suggests that longer copulations facilitate ejaculate transfer and lead to increased female lifetime fecundity (see e.g. [23]). In several species, sperm is known to be transferred only at the very beginning of copulation, while during the later stages of copulation males transfer substances that build effective mating plugs or other manipulating seminal fluids [24,25]. The 'extended mate guarding hypothesis' posits that with longer mating, the chances of the female being inseminated by a subsequent male are reduced [9,22,26]. Prolonged copulation durations have been found to act directly as mate guarding (reviewed in [26]). To understand the adaptive significance of prolonged copulations, it is necessary to identify which sex controls the duration of copulation. Among *Drosophila *species, copulation duration has been found to be largely under male control in *D. melanogaster*, *D. simulans*, *D. mojavensis *and *D. athabasca *[27-31] or determined through interactions between males and females in *D. elegans *[32]. A recent study of the functional significance of external genital structures in *D. melanogaster *and *D. simulans *[33] confirmed that females are coerced into mating for as long as suits the males' interests. Here, the male control relies on a 'lock'-like genital coupling mechanism and females are unable to break the lock without sustaining harm. While there is ample evidence for anatomical modifications enabling males to coerce females into mating, so far no indication has been found in Drosophilidae of modifications of the female genitalia that may have evolved to defy male control, as documented in other insects [34]. In general, up to date, reports of (at most partial) female control over copulation duration are sparse (e.g. in *D. mojavensis *[30]). The ejaculate transferred to females consists not only of sperm but also seminal fluids. In several species, seminal fluids have been shown to contain substances that manipulate female physiology and behaviour [35]. Possible manipulations e.g. in *D. melanogaster *[36] include prolonged latency to remating or preventing remating altogether, decrease of subsequent copulation durations, enhanced reproductive output, sometimes at the females' expense by derogating longevity, alteration of immune response and feeding behaviour, increased storage and utilization of sperm.

Accessory gland proteins (acps) most likely evolved to stimulate reproductive processes in order to synchronise female receptivity with male reproductive effort. However, these substances can be deleterious in high doses [37] and males can utilize them to manipulate female physiology and behaviour to their advantage. Seminal fluid proteins evolve much faster than non-reproductive proteins, providing indirect evidence for sexual selection or an underlying arms race of sexual antagonistic co-evolution between male coercion and female resistance (see [4] for a review on acps and sexual conflict in *Drosophila*).

In *Drosophila montana*, a promiscuous species in the large *Drosophila virilis *species group, males and females apparently undergo a conflict of interest during copulation. Once a female has allowed a courting male to mount, and after an initial phase of apparent harmony, copulating pairs enter an obvious conflict phase, where females conspicuously attempt to dislodge the mounting male by vigorously kicking with their hind legs, wing flicking or decamping. While there is good knowledge of sexual selection processes in *D. montana *and other species of the *D. virilis *species group (reviewed in [38]), little is known about the potential for sexually antagonistic co-evolution in this group. Our aim was to explain the males' efforts to prolong matings beyond the females' preferred duration. First, we tested whether copulation duration is a form of reproductive investment that responds to variation in the OSR as predicted by sperm competition game models. Second, we investigated which of the sexes exerts control over the duration of copulation by comparing the duration of copulation of unmanipulated females and of females whose resistance attempts were precluded. Third, we addressed potential costs arising from prolonged copulations to females and potential benefits accruing to perseverant males, in terms of reproductive output and in terms of the share of paternity in the face of competition against a later rival.

## Results

### Sex ratio manipulation

Across matings, the durations of the struggle phase and of the entire copulation were significantly positively correlated (N = 115, r = 0.56, P < 0.001), indicating that perseverant males indeed obtained increased mating time. The length of copulation until the onset of female reluctance and the length of the struggle were significantly negatively correlated (N = 115, r = -0.22, P = 0.017). Hence, the earlier on males were rejected, the longer they struggled to hold on. Conversely, males that had been tolerated for longer already soon gave up when rejected, or even disengaged on their own accord.

**Figure 1:**
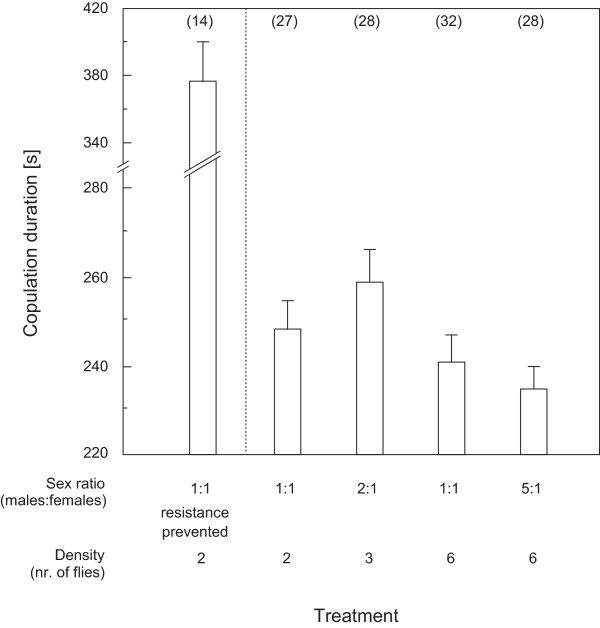
**Duration of copulations with prevented female resistance and under different sex ratios**. The bar to the left of the dotted line shows copulation duration in seconds when females were prevented from dislodging the mounting male (mean ± SE, note the broken scale on the y-axis), bars to the right of the dotted line show duration of copulation (mean ± SE) for different sex ratio manipulation treatments. Sample sizes are given in parentheses.

In agreement with theoretical predictions, matings were longest in the presence of two males contending for a single female, shortest when five males were competing, and intermediate at even sex ratios (Figure 1, bars to the right of the dashed line). Even though conventional (non-directional) analysis of variance failed to reveal significant effects of the sex ratio manipulation, the means of copulation duration precisely matched our a priori ordered hypothesis (one-way ANOVA, F_3,111 _= 2.09; OH-test, r_s_P_c _= 0.85, P_dir _< 0.01). Alternative hypotheses linking the duration of matings and struggles with the total density of individuals, or with the density of one sex (e.g. because of changes in the availability of potential mates or in the occurrence of intrusions) are not supported by the observed response pattern.

### Control over copulation duration

When females were prevented from dislodging the copulating male, matings lasted one and a half times longer than when female movement was unconstrained (mean ± SD 377 ± 90 s versus 248 ± 38 s, t = 6.58, d.f. = 39, P < 0.001, two leftmost bars in Figure 1). Hence, female reluctance behaviour effectively contributes to reducing copulation durations.

### Mating interruptions

**Figure 2:**
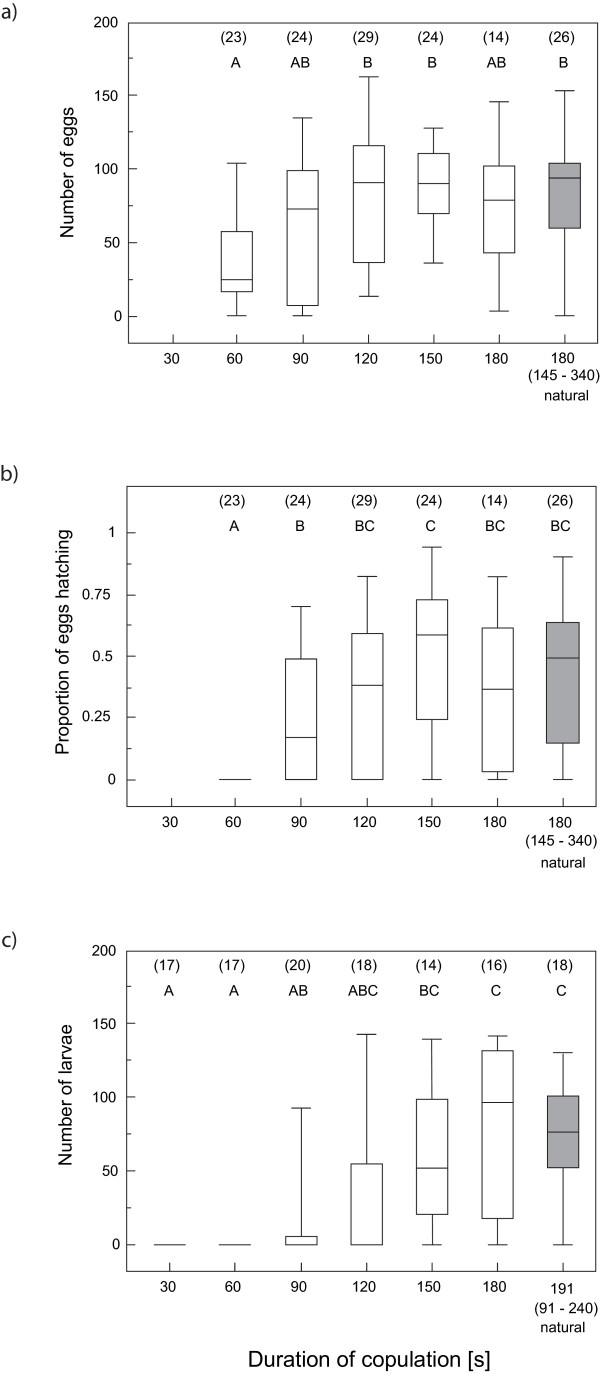
**Female reproductive output depending on copulation duration**. Female reproductive output in terms of (a) egg production, (b) egg hatching success, and (c) larvae production for matings interrupted at predetermined times (white boxes) and naturally terminated matings (shaded box). The box plots represent the median as the middle line, 25 and 75% quantiles as box boundaries, and 10 and 90% quantiles as whiskers. The median (range) duration of copulation for unmanipulated matings is given under the corresponding box. Classes not sharing the same letter are significantly different (all H > 23.6, all P < 0.01, from a Kruskal-Wallis ANOVA followed by Tukey-type multiple comparisons). Sample sizes are given in parentheses.

The reproductive output in terms of egg production, egg hatching success and larvae production of females whose matings were interrupted at predetermined times showed an overall positive relationship with copulation duration, up to a certain plateau (Figures 2a–c). None of the matings interrupted within one minute of initiation produced any offspring. Female productivity rose steeply during the second minute into copulation but soon levelled off, such that not significantly fewer eggs or progeny were produced in matings interrupted two minutes after their commencement than in naturally terminated matings (mean ± SE of naturally terminated copulations combined from egg count and larvae count 193.5 ± 5.1 s).

Within groups of females that were allowed to terminate matings naturally, progeny production bore no relationship to copulation duration (linear and quadratic fits, all R^2 ^< 0.07, all P > 0.3). One out of 26 unmanipulated matings in the egg production and hatching success assay, and two out of 18 unmanipulated matings in the larvae production assay yielded no eggs, giving an estimate of female infertility rate of ca 4 to 11%. For comparison, ca 3% of the females whose mating was interrupted (one after 60 s and two after 90 s, out of 114 total) did not lay any eggs (calculated from the egg production assay sample). Three of the 26 unmanipulated matings that did yield eggs did not result in viable offspring, providing an estimate of male sterility of ca 12%, possibly inflated by environmental causes of egg mortality.

**Figure 3:**
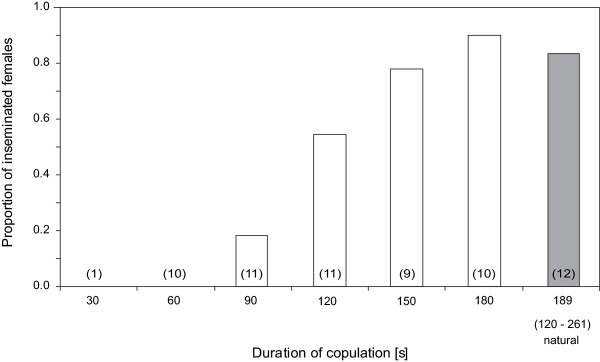
**Proportion of inseminated females depending on copulation duration**. Proportion of inseminated females for matings interrupted at predetermined times (white bars) and naturally terminated matings (shaded bar). The median (range) duration of copulation for unmanipulated matings is given under the corresponding bar. Sample sizes are given in parentheses.

The dynamics of sperm transfer closely matched the pattern inferred from the female productivity data. No sperm was recovered from females whose matings lasted less than one minute. The proportion of inseminated females asymptotically increased in subsequent intervals to reach a plateau at around 150 s, after which time 90% to 100% of all females were inseminated (non-linear logistic regression on ln-transformed copulation durations, Wald = 17.6, P < 0.001, with 80% correctly classified cases, Figure 3). Accordingly, inseminated females experienced significantly longer copulations than non-inseminated ones (Wilcoxon rank sum test, N_inseminated _= 26, N_non-inseminated _= 29, Z = -4.64, P < 0.001).

### Double matings

#### Female remating

**Figure 4:**
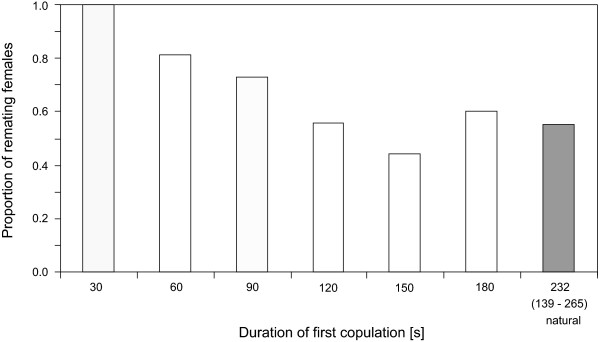
**Proportion of females that remate depending on duration of first copulation**. Proportion of remating females when first matings had been interrupted at predetermined times (white bars) or allowed to be terminated naturally (shaded bar). The median (range) duration of copulation for unmanipulated first matings is given under the corresponding bar.

All females that were allowed only 30 s for the first copulation remated in the remating experiment. Then the proportion of remating females dropped in each of the time classes to about one half in the class where the first copulation was interrupted after 120 s. The remating rate remained largely unchanged for first copulations that lasted longer than 120 s (Figure 4). Accordingly, females that remated had experienced significantly shorter first copulations than females that did not remate (Wilcoxon rank sum test, N_remated _= 109, N_non-remated _= 60, Z = 3.58, P < 0.001).

**Figure 5:**
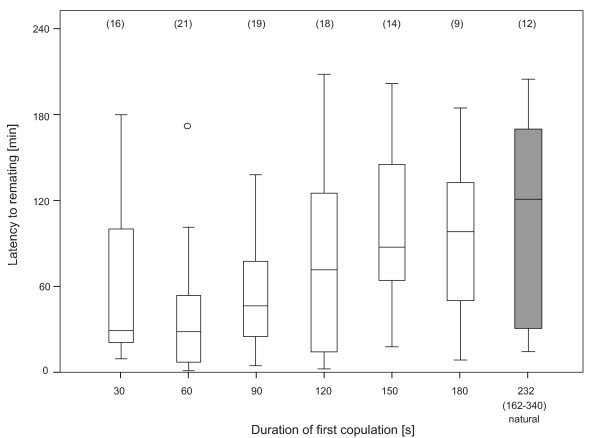
**Latency to remating depending on duration of first copulation**. Latency to remating (in minutes from introduction of the second male until copulation) 24 h after first matings that were either experimentally interrupted (white boxes) or naturally terminated (shaded box). The box plots represent the median as the middle line, 25 and 75% quantiles as box boundaries, and 10 and 90% quantiles as whiskers. Sample sizes are given in parentheses.

Females that did eventually remate did so faster, the sooner their first copulation had been interrupted (logistic regression, Wald = 11.8, P = 0.001, with 63% correctly classified classes, Figure 5). Latency to remating was significantly shorter when the first copulation had been interrupted after 60 s than when the first copulation was terminated naturally (mean difference 68 ± 20 min, P = 0.016) or interrupted after 150 s (60 ± 19 min, P = 0.035). A trend is still present in the 90 s class as compared to naturally ended first copulations (56 ± 20 min, P = 0.099; overall model ANOVA on square root transformed latencies, F_6,108 _= 3.42, P = 0.004, significance of mean differences between groups determined with Tukey HSD posthoc tests).

#### Paternity analyses

**Figure 6:**
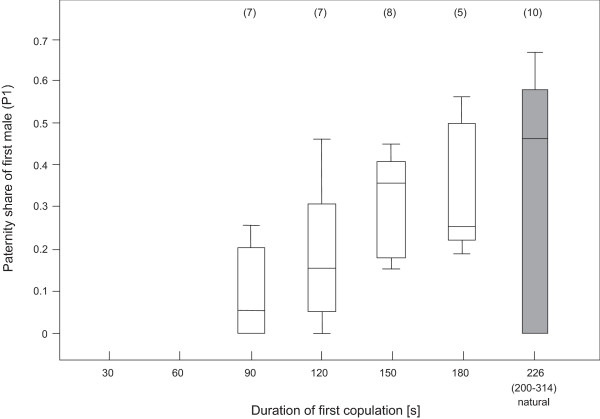
**Proportion of progeny sired by the first male to mate depending on duration of first copulation**. Proportion of progeny sired by the first male to mate (P_1_) when first matings were interrupted at predetermined times (white boxes) or allowed to be terminated naturally (shaded box) and second matings were allowed to be terminated naturally. The median (range) duration of copulation for unmanipulated first matings is given under the corresponding box. Sample sizes are given in parentheses.

In remating experiments where the first mating was interrupted while the second was allowed to be completed, the proportion of progeny sired by the first male relative to a subsequent rival (P_1_, a defensive measure of sperm competition) increased with the duration of the first mating (Figure 6). However, the paternity share of the first male (corrected for the confounding effect of the duration of the second copulation by using the regression residuals for analysis) rose only up to a copulation duration of about 120 s and remained stable thereafter at the level of naturally terminated matings (ANOVA F_4,36 _= 2.97, P = 0.034, Dunett's t posthoc test: naturally terminated control copulations were used as control against which all other groups were tested. 90 s mean difference in paternity of P_1 _to naturally terminated control group -25.3%, P = 0.013; 120 s to control -18.8%, P = 0.068). Conservatively, the comparison neglects females that did not remate (i.e. strictly, P_1 _= 1), which were often precisely those experiencing the longest copulations. Similarly conservatively, we did not determine P_1 _for pairs with copulation durations shorter than 90 s, because of previous indications that no sperm is transferred within such a short time frame and thus that the second male to mate would sire all of the produced offspring (P_1 _= 0).

## Discussion

### Sex ratio manipulation

Male *D. montana *gauged the intensity of competition from rival males and adjusted the duration of copulation, used as a proxy of reproductive investment, accordingly. Copulations were longest in the presence of one potential rival, shortest in the presence of four potential rivals, and intermediate in the absence of competition. The pattern thus precisely matches theoretical predictions based on diminishing returns per unit of expenditure with increasing competition [17]. Parker et al.'s [17] evolutionary stable strategy (ESS) models were originally derived to determine optimal sperm expenditure in external spawners, but they have been equally applied to internally fertilizing species in which ejaculates compete in a fair raffle, i.e. in which fertilization success is proportional to the number of sperm transferred [20,39]. When the raffle is loaded, i.e. when one male's sperm is devalued relative to the sperm of another male, the ESS depends on the information the parties have about their role, and whether or not their roles are occupied randomly [40]. In *D. montana*, the raffle is loaded in favour of the last male (see Results, also [41]). All our experimental males housed for the time of the experiment with one or more rivals mated under the overt threat of not being the last male to mate. While we used only virgin females in our study, in nature most matings will involve non-virgins, and most males will not be their partner's last mate. However, copulation durations in our double mating experiments suggest that the ability of *D. montana *males to distinguish virgin from mated females is at most imperfect.

A few studies have addressed the question of how flexible the response to changes in OSR (operational sex ratio) is. OSR can fluctuate substantially in both space and time [42], and the individual ability to promptly track changes in OSR would optimize the adjustment of investment to the prevailing circumstances [43-45]. For *D. montana *males used in our sex ratio manipulation, the effect of ambient OSR overshadowed the effect of the premating OSR (all males having spent practically their entire adult life in single-sex groups). Species of the *D*. *virilis *group have a short reproductive season in spring, during which flies of both sexes converge on food patches of rotting plant material to mate [46,47]. Under such a 'sex at the restaurant' scenario, it makes adaptive sense to respond to the current OSR, rather than to the OSR experienced beforehand elsewhere.

### Control over copulation duration

In contrast to earlier reports that copulation duration is ultimately under the control of males in various *Drosophila *species [27-31], we showed that *D. montana *females make a substantial contribution towards shortening the duration of copulation. When female resistance attempts were suppressed, males persisted in copula far longer than they ever managed to in unmanipulated matings. To a human observer, the females' desperate efforts to shake off the mounted male appear to reflect a premium for keeping matings within a certain optimal time frame or, conversely, a cost from matings that last longer than required from their own perspective.

### Costs and benefits of prolonged copulation durations

We explicitly addressed and documented a potential cost accruing to females coerced into exceedingly long matings, relative to their preferred duration. The longer the females' first copulation, the lower the proportion of females that remated on the following day, and the longer the remating latency for eventually remating females. Males clearly profit from a delay in subsequent female remating through the extended period during which their sperm is used exclusively to sire progeny (see also [48]). Nearly all females were inseminated well before the end of naturally terminated copulations. Thus, the 'ejaculate transfer hypothesis', which postulates that copulation duration and sperm transfer/storage are positively correlated, does not fit the observed pattern as well as the 'extended mate guarding hypothesis'. The latter hypothesis provides here a better explanation, as the prolonged remating latency in females after longer copulations reduces the likelihood of females being inseminated by a subsequent male. The onset of female reluctance behaviour after sperm transfer seems to have reached its maximum suggests that males avail themselves of proteins in the seminal fluid to modify female behaviour to their advantage, as has earlier been shown in other species [36,49]. Also, the fact that females produced eggs after copulations lasting less than required for sperm transfer to occur (90 s) might be attributed to the action of fecundity-enhancing seminal fluids transferred at the very beginning of copulation, even before sperm transfer begins. Even though an induced refractoriness can under certain circumstances be beneficial to females [50], it turns detrimental when it drives female mating rate away from the optimum, and obviously compromises female interests when it curtails the benefits to be derived from multiple mating.

Arnqvist and Nilsson [50] provide convincing arguments for the claim that a single mating does not in general maximize lifetime fitness of female insects. Even in species without nuptial feeding, direct benefits of remating in the form of replenished sperm supplies, stimulating or gonadotropic effects of mating, and nutritional or hydrating effects of male ejaculate substances more than offset negative effects for moderate remating rates. Another direct benefit of multiple matings for females lays in counteracting male sterility, which can be as substantial as the approximate 12% we estimated in male *D. montana*. Additional indirect benefits accruing to females from mating with different partners (i.e. polyandry) such as increased offspring diversity and the opportunity for bet-hedging against genetically incompatible or inferior males [51,52] further promote and maintain female remating behaviour. Aspi's [53] survey of a natural population of *D. montana *in Finland revealed that virtually all sampled females carried sperm of at least two males. In the field, copulations were commonly observed posterior to the date when 95% of all the females were inseminated [47]. In the laboratory, females readily remate when given the opportunity, some within minutes, and ca 40% within one day ([41] and personal observations). As female cooperation is an essential prerequisite for copulation to occur in *D. montana *[54], remating is likely an adaptively favoured female strategy, rather than an inability to physically resist coercive manipulation. Whether the reported delay in remating associated with longer first copulations imposes a significant fitness penalty is an issue that merits further attention, as is the investigation of other potentially incurred costs in terms of lifetime fecundity and of survival.

## Conclusion

While male interests in sexual and sexually antagonistic selection scenarios have been studied intensively and are rather straightforward, females' interests have only lately come under scrutiny. The fact that the general effect is notoriously difficult to gauge in females (see chapter 6 in [26]) may account for this delay in interest. Our study provides empirical evidence for substantial female control over copulation duration in a *Drosophila *species. We also show for the first time in a *Drosophila virilis *group species (a species that has lately been paid much attention because of its suitability for studying the genetic basis of co-evolving sexual behaviour) that the main cost to females and fitness benefit to males of prolonged copulations lay in the extended latency to female remating, leading to a conflict of interest between the sexes.

## Methods

### General methods

Flies used in experiments were collected from replicate culture plexiglas vials of three isofemale lines (strains) from Yukon (Alaska, USA, 61°30'N, 159°20'W), Oulanka (Finland, 66°25'N, 29°0'E) and Kawasaki (Japan, 34°80'N, 139°60'E) which had been inbred in the laboratory for over twenty generations (OSR and copulation duration experiments), and from isofemale lines established from collections made in 2003 in the surroundings of Vancouver (British Columbia, Canada, 49°16'N, 122°55'W, all other experiments). Experiments were conducted at the Universities of Jyväskylä, Finland (egg counts, hatching success, parentage analysis) and St Andrews, Scotland (OSR manipulation, larvae counts, sperm counts). The two laboratories have established identical methods, conditions and recipes, and have access to the same fly strains.

Strains were maintained at a density of approximately 50 flies per vial on malt medium sprinkled with live yeast and housed under constant conditions at a temperature of 19 ± 0.5°C with continuous light. Males and females were collected within three days of emergence to ensure virginity. The virgin flies were sorted into separate sexes under light CO_2 _anaesthesia and placed in vials in single sex batches of five or six. The day preceding their use in experiments, males and females were isolated into individual vials. All flies were used upon reaching sexual maturity (age 21 ± 2 d). Experimental matings were staged in food vials (height: 8 cm, diameter 3 cm) stood upright and held at room temperature (18 ± 1°C) under continuous lighting provided by light bulbs (11W/827) suspended ca 40 cm above the vial rims.

### Sex ratio manipulation

To examine whether males adjust the time spent in copula according to the perceived presence of potential competitors, we recorded the duration of matings under different operational sex ratio regimes. Virgin males and females were randomly allocated to one of four treatments, differing in the intensity of competition among males for access to females: (1) one female and one male, (2) three females and three males (3) one female and two males, and (4) one female and five males. The treatment consisting of groups of three females and three males was intended to partially control for the increased disturbance ensuing from higher densities expected in the treatment with the most skewed male-bias, while keeping an even sex ratio. However, typically, copulating couples were ignored by the other flies. Occasionally, one rival male would display close to the mating pair or try to mount the mating male. We never witnessed a successful take-over attempt. If anything, the other males would court the still available females (in the three males and three females treatment) or court each other (in the one female and five males treatment) in male-male chains, as described for some *D. melanogaster *mutants [55]. Females were placed into vials first, and then the appropriate number of males was added. Flies were handled without anaesthesia. We recorded the duration of copulation from when the genitalia first locked until the male dismounted. We noted the time of the onset of the female kicking behaviour and calculated the length of the struggle between partners. In the treatment involving groups of three females and three males, we considered the first copulation to occur. Only males and females from Finland and Japan were used. The four resulting strain combinations (male or female from Finland or Japan) were evenly distributed across treatments. Up to eight vials were watched simultaneously, although no more than three replicates per treatment ran concurrently at any one time. All females were used only once, regardless of whether they mated or not. Males from vials in which no copulation occurred within four hours were re-tested on the following day, in the same group and treatment. In between observations, such males were held individually in fresh food vials. Whether males were used for the first or the second time did not significantly affect their copulation duration (P > 0.5). Groups of males failing to obtain any copulation within two observation sessions on subsequent days were discarded. An insect pin stuck in the medium was added in the treatment with one single pair to provide the suitable control for the manipulation where female resistance was prevented (see section 'Control over copulation duration' below).

### Control over copulation duration

In order to investigate which of the sexes holds control over the duration of copulation, we measured the length of copulations in which the females' attempts to dislodge their mates were precluded. Only males and females from the two strains Finland and Japan were used in this experiment, whereby the four strain combinations (male or female from Finland or Japan) were similarly represented. Females were killed by an overdose of CO_2 _and then carefully pinned through the thorax, slightly to the right of the midline (Karlsbader insect pins nr. 0, Ernst Wirkner, Germany). The pin was then stuck into the malt medium layering the bottom of a food vial. We used fine forceps to adjust the inclination of the body and the spreading of the wings in a fair imitation of the typical wing lifting posture with which *D. montana *females signal readiness to mate [56], and to which males invariably respond with mounting [57]. Occasionally, when the male vigorously courted the pinned female, showing an obvious willingness to mate, we removed the cotton wool plug sealing the vial and gently nudged the pinhead. Slight movement of the pinned dead female occasionally helped to persuade reluctant males to mount. Trials were watched continuously for up to four hours or until copulation occurred. Males that did not mate within four hours were reused up to two times on subsequent days. We recorded the duration of copulation (in seconds) from the time of intromission until the male withdrew its aedeagus and dismounted.

### Mating interruptions

Experimental interruptions of matings at predetermined times were conducted to examine whether males that protract matings beyond the females' preferred duration are rewarded by increased progeny production. One virgin female and one virgin male were placed together in a food vial. Flies were handled without anaesthesia. We ensured that all vials contained similar amounts of malt medium. Vials were scanned continuously until copulation occurred. We noted copulation start as the time when the genitalia were first observed to lock. Pairs were randomly assigned to different copulation duration treatments. After either 30, 60, 90, 120, 150 or 180 s, matings were interrupted by gently aspirating the pair up and down a thin rubber hose. Timings were accurate to within ten seconds. We had to forgo planned additional time intervals of 210 s and 240 s, as most copulations were naturally terminated beforehand. Females that started resisting males ahead of their set time were omitted from all analyses. In an identically handled control group, matings were allowed to proceed undisturbed until naturally terminated. All males and females were used only once. Immediately after the end of copulation (forced or spontaneous), males were removed to prevent remating. Females whose productivity was to be assessed were left to oviposit in the vials for three days, after which they were moved to fresh vials to avoid excessive larval crowding. Once-mated *D. montana *females produce progeny for about six days [53], though most (fertilized) eggs are laid on the second to fourth day following insemination (own observation). The experiment was repeated three times, firstly to determine egg production and hatching success, secondly to determine offspring production by counting larvae and thirdly to estimate the amounts of transferred sperm per time unit.

#### Egg production and egg hatching success

Egg production and hatching success were determined for females that had been allowed to copulate for predetermined durations (14 to 29 females per time interval), and for 26 females that terminated copulations naturally. Eggs laid in the first six days following mating (into two vials per female) were counted under a binocular microscope. Eggs can easily be spotted as circular whitish dots on the surface of the medium. The sum of the number of eggs in the two vials of each female was used for analyses. Vials were then kept under the same conditions as described above for general fly maintenance. All adults emerging from those eggs were counted, and the proportion of hatched eggs was calculated (hatching success, i.e. survival to adulthood).

#### Larvae production

Larvae production was determined 12 ± 1 days following matings of predetermined duration for 14 to 20 females per time interval, and for 18 control females allowed to terminate copulation naturally. The upper layer of the medium, which contained all of the (second- and third-instar) larvae, was dissolved in warm water and dispersed on filter paper. Larvae were killed by drying the filter paper in a drying cupboard (ca 50°C) and then counted under a binocular microscope.

#### Sperm transfer

As in the above assays no progeny were produced in copulations lasting less than 90 s, the question arose of whether any sperm at all was transferred or rather whether sperm was transferred but not utilized by the females during the earlier stages of copulation. For assessment of sperm transfer, the earliest interruption was made after 60 s. Ten to eleven pairs were tested in each time interval plus twelve pairs that were allowed to end copulation naturally. Immediately after the copulation was either experimentally or naturally terminated, the females were dissected under a deep CO_2 _anaesthesia in a standard insect ringer solution (PBS buffer). Sperm in uterus, spermatheca and receptaculum seminis was scored as present or absent, as sperm migration is easily detected in non-dyed tissues. This rather crude method was applied because methods to quantify the amount of transferred sperm failed, most likely due to sperm morphology: *D. montana *has very long spermatozoa of ca 3 mm (for comparison: male thorax length ca 1.5 mm [58,59]), which could not be straightforwardly extracted from the reproductive organs of the females. Attempts to dye the sperm heads with DNA-specific fluorescent dyes within the female reproductive tract to count transferred sperm failed.

### Double matings

We conducted a double-mating experiment to test whether males that coerce females into protracted matings derive an advantage in the competition for paternity against a later rival. Females were mated twice on consecutive days with different males. Only Canadian strains were used. Males were chosen from two strains fixed for different-sized alleles at the microsatellite locus *Mon5 *(described by [60]), hence allowing unambiguous assignment of paternity of all offspring within a clutch with the use of only one marker. Male strains were randomly assigned to mate first or second. First matings were interrupted after either 90, 120, 150 or 180 s, or were left to end naturally.

#### Female remating

Females were remated on the following day. Latency to remating was measured as the time interval between the time of introduction of the second male and the start of remating. Females that refused their suitors' advances for up to four hours were scored as unwilling to remate. Females that were not courted by their assigned second partner were omitted from analyses. All of the second matings were allowed to proceed until naturally terminated. Males were stored in 70% ethanol for molecular analyses. Females were given a 9-day egg laying period and were then discarded. Upon emergence, 16–20 offspring per female were collected for DNA extraction and paternity analyses.

#### Paternity analyses

Genomic DNA from single flies was isolated by the "single fly DNA preps" method [61]. The microsatellite locus *Mon5 *[60] was amplified in 10 μl reactions containing 50–100 ng of genomic DNA, 1 μM of each primer (TAGC, Copenhagen, Denmark), 200 μM dNTPs, 1.5 mM MgCl_2 _and 1 unit *Taq *DNA polymerase (Biotools B&M Labs, Madrid, Spain) in 1× manufacturer's buffer. The reaction profile included denaturation at 94°C for 3 min, then 94°C for 50 s, T_a_°C for 50 s, 72°C for 50 s for 34 cycles, then a final extension at 72°C for 4.5 min. T_a_, the annealing temperature, was initially set to 4°C higher than 63°C as given in [60] and dropped by 1°C in each of the four initial cycles (from 63°C to 59°C) to increase primer specificity. PCR amplification was performed on an MBS Satellite 0.2 G Thermal Cycler (Thermo Electron, Milford, MA, USA). The forward primers were fluorescently labelled for automated fragment sizing of PCR products run on an ABI Prism 3100 Sequencer (Applied Biosystems, Foster City, CA, USA) using GeneMapper v.3.7 (Applied Biosystems, Foster City, CA, USA).

### Statistical analyses

Data analysis was performed using JMP 7.0 for the Mac and SPSS 15.0 for Windows. Normality and homogeneity of variances of all raw data and residuals from models were checked by Shapiro-Wilk and Bartlett's tests, respectively.

#### Sex ratio manipulation

Copulation durations (square root transformed to meet parametric assumptions) were compared using an ANOVA model with the fixed factor treatment (sex ratio manipulation). Optimality models [17,62] provided an explicit ordered prediction of the effect of our sex ratio manipulation on the duration of copulation, thus justifying the examination of statistical tests for ordered alternative hypotheses, i.e. ordered heterogeneity (OH)-test [63] in addition to conventional unordered statistics. The OH-test incorporates the Spearman rank correlation (r_s_) between the observed and the expected orders in the calculation of a new test statistics P_c_r_s_, where P_c _is the complement of the probability value from the conventional non-directional test (P_c _= 1- P). P_c_r_s _combines the magnitude information in the sample extracted by P_c _(the heterogeneity component) with the independent information extracted by r_s _(the ordering component). It thereby allows not only a test against the null hypothesis of no differences between treatments, but, additionally, to refute the null hypothesis in the direction of an anticipated alternative hypothesis. Given corresponding probability values are read from a graph (Figure 1 in [63]), and hence approximate.

#### Mating interruptions

Because of many unproductive interrupted matings, distributions were often skewed such that transformations failed to normalize the data. Data are thus analyzed using Kruskal-Wallis analysis of variance (ANOVA) followed by Tukey-type multiple comparisons after Zar [64] calculated by hand and verified using SsS 1.1a (Rubisoft Software GmbH, Eichenau, Germany).

## Authors' contributions

DM and KK conceived of the study, designed all experiments and performed some of them, conducted the statistical analyses and wrote the manuscript. JK performed some of the experiments and all molecular analyses, and participated in data analysis and in drafting the manuscript. AH participated in the design and coordination of the study, and discussed its interpretation and presentation. All authors read and approved the final manuscript.
